# Roles of RNA-Binding Proteins in DNA Damage Response

**DOI:** 10.3390/ijms17030310

**Published:** 2016-02-27

**Authors:** Mihoko Kai

**Affiliations:** Department of Radiation Oncology, Johns Hopkins University, School of Medicine, Baltimore, MD 21231, USA; mkai2@jhmi.edu; Tel.: +1-410-614-9223

**Keywords:** DNA damage, DNA repair, RNA-binding proteins, intrinsically disordered proteins, prion-like domain, FUS, RBM14

## Abstract

Living cells experience DNA damage as a result of replication errors and oxidative metabolism, exposure to environmental agents (e.g., ultraviolet light, ionizing radiation (IR)), and radiation therapies and chemotherapies for cancer treatments. Accumulation of DNA damage can lead to multiple diseases such as neurodegenerative disorders, cancers, immune deficiencies, infertility, and also aging. Cells have evolved elaborate mechanisms to deal with DNA damage. Networks of DNA damage response (DDR) pathways are coordinated to detect and repair DNA damage, regulate cell cycle and transcription, and determine the cell fate. Upstream factors of DNA damage checkpoints and repair, “sensor” proteins, detect DNA damage and send the signals to downstream factors in order to maintain genomic integrity. Unexpectedly, we have discovered that an RNA-processing factor is involved in DNA repair processes. We have identified a gene that contributes to glioblastoma multiforme (GBM)’s treatment resistance and recurrence. This gene, *RBM14*, is known to function in transcription and RNA splicing. *RBM14* is also required for maintaining the stem-like state of GBM spheres, and it controls the *DNA-PK*-dependent non-homologous end-joining (NHEJ) pathway by interacting with *KU80*. *RBM14* is a RNA-binding protein (RBP) with low complexity domains, called intrinsically disordered proteins (IDPs), and it also physically interacts with PARP1. Furthermore, *RBM14* is recruited to DNA double-strand breaks (DSBs) in a poly(ADP-ribose) (PAR)-dependent manner (unpublished data). DNA-dependent *PARP1* (poly-(ADP) ribose polymerase 1) makes key contributions in the DNA damage response (DDR) network. *RBM14* therefore plays an important role in a *PARP*-dependent DSB repair process. Most recently, it was shown that the other RBPs with intrinsically disordered domains are recruited to DNA damage sites in a PAR-dependent manner, and that these RBPs form liquid compartments (also known as “liquid-demixing”). Among the PAR-associated IDPs are *FUS/TLS* (fused in sarcoma/translocated in sarcoma), *EWS* (Ewing sarcoma), *TARF15* (TATA box-binding protein-associated factor 68 kDa) (also called FET proteins), a number of heterogeneous nuclear ribonucleoproteins (*hnRNP*s), and *RBM14*. Importantly, various point mutations within the *FET* genes have been implicated in pathological protein aggregation in neurodegenerative diseases, specifically with amyotrophic lateral sclerosis (ALS), and frontotemporal lobe degeneration (FTLD). The FET proteins also frequently exhibit gene translocation in human cancers, and emerging evidence shows their physical interactions with DDR proteins and thus implies their involvement in the maintenance of genome stability.

## 1. Introduction

Involvements of RNA-binding proteins (RBPs) in DNA damage response (DDR) have been described, and growing evidence suggests their roles in DNA repair. Large-scale proteomic and genetic screenings have identified RNA processing factors including RBPs in major functional categories of *ATM* (ataxia-telangiectasia mutated)/*ATR* (ataxia telangiectagia and Rad3 related) substrates [1] and in the prevention of genomic instability [2,3]. However, mechanisms of their involvement in DDR regulation remain elusive. Recent findings on liquid demixing produced by poly(ADP-ribose) (PAR)-dependent recruitments of RBPs shed new light on how DDR might be initiated.

## 2. Control of DNA Damage Response (DDR)-Gene Expression by RNA-Binding Protein (RBP)

A large body of evidence suggests that RNA-processing factors regulate the expression of several genes involved in DDR. For example, one of the FET proteins in Ewing sarcoma (*EWS*) depletion results in alternative splicing (AS) changes of genes involved in DNA repair and genotoxic stress signaling, including *ABL1, CHK2*, and *MAP4K2.* Chromatin and RNA crosslinking immunoprecipitation experiments show that *EWS* co-transcriptionally binds to its target RNAs. This association is decreased after UV irradiation of cells, concomitant with transient enrichment of EWS in nucleoli and with AS changes that parallel those induced by *EWS* depletion that lead to reduced *c-ABL* expression [4].

*BRCA1* (breast cancer 1), one of the key players in the cell cycle checkpoint and homologous recombination (HR), interacts with the mRNA splicing factor *BCLAF1*. This *BRCA1-BCLAF1* complex regulates mRNA splicing of DDR genes *ATRIP*, *BACH1*, and *EXO1* in response to DNA damage, although AS of these genes were not detected in this study. Importantly, *ATM/ATR*-dependent phosphorylation of *BRCA1* at Ser1423 is required for its interaction with *BCLAF1* [5].

Levels of the tumor suppressor and the DNA damage checkpoint protein *p53* are increased in response to DNA damage [6]. The *p53* protein level is primarily regulated through ubiquitin-mediated proteolysis, and DNA damage-induced phosphorylation inhibits this degradation process [7]. However, *p53* mRNA stability and translation are shown as strong regulators of *p53* expression [8]. The RNA-binding protein *HuR* (Hu antigen R) binds to *p53* mRNA and enhances its translation [8]. In response to ionizing radiation (IR)-induced damage, *HuR* targets mRNAs that encode DDR-, apoptosis-, and proliferation-related proteins, such as *53BP1*, *MDM2*, *BAX*, *K-Ras*, and *p21* [9,10,11]. Importantly, one of the DNA damage checkpoint kinases, *CHK2*, phosphorylates *HuR* in response to DNA damage, and the *CHK2*-dependent phosphorylation induces *HuR*’s association and dissociation from its target mRNAs. *CHK2* phosphorylation-induced dissociation of *HuR* is import for cell survival after IR exposure [10]. It is also required for regulation of the mRNA expression encoding the longevity and stress-response protein *SIRT1* [12]. *HuR* binds to *SIRT1* mRNA and stabilizes it, leading to increased *SIRT1* expression levels. Oxidative stress triggers the dissociation of *HuR* from *SIRT1*, promoting *SIRT1* mRNA decay. The influence of *HuR* function by *ATM*-*CHK2*-dependent phosphorylation underscores the intricate connections between the RBP and DNA damage and stress responsiveness [12].

The RNA-binding protein *RBMX* has been identified as a protein involved in DDR by a genome-wide siRNA screen to find components of the mammalian HR machinery using a well-characterized GFP (green fluorescent protein)-based HR reporter [13]. *RBMX* is a heterogeneous nuclear ribonucleoprotein that has a role in alternative splicing [14]. Knockdown of *RBMX* inhibits HR, and *RBMX* accumulates at DNA lesions through multiple domains in a *PARP1*-dependent manner. However, *PARP1* inhibition does not cause HR defects, and inhibition of HR by *RBMX* knockdown seems to be caused by the reduced expression of *BRCA2*. Similar effects have been observed with knockdown of some of the other pre-mRNA-processing genes that have been identified by the screening [13].

## 3. Direct Roles of RBPs in DDR

In addition to their roles in AS and the expression of DDR genes, direct involvement of RBPs in DDR has been shown. *PRP19* is an ubiquitin ligase and an important regulator of pre-mRNA splicing [15,16,17]. During splicing, *PRP19* ubiquitylates the U4 small nuclear ribonucleic particles (snRNP) component *PRP3*, leading to the stabilization of the *U4/U6.U5 snRNP* [18]. *PRP19* interacts with RNA polymerase II, and couples RNA processing and transcription [19,20]. Interestingly, yeast *PRP19* (also called *PSO4*) has been identified by independent genetic screenings for splicing as well as DDR mutants [21,22,23]. It has also been shown in human cells that *PRP19* interacts with *WRN* (Werner syndrome, RecQ helicase-like), which has DNA helicase and nuclease activities, for the processing of DNA interstrand cross-links and with terminal deoxynucleotidyl transferase for DNA repair [24,25]. A core component of the putative E3 ubiquitin ligase complex of *PRP19*, *CDC5L*, interacts with *ATR*. *CDC5L*-depleted cells are sensitive to replication blocking agents, and are defective in S-phase checkpoint. Furthermore, *CDC5L* is required for the activation of downstream effectors or mediators of *ATR* checkpoint function, such as *CHK1*, *RAD17*, and *FANCD2* proteins [26]. Recent proteomic screenings have identified *PRP19* as a protein that interacts with *RPA* (replication protein A)-coated single-stranded DNA (ssDNA) [27,28]. *PRP19* binds to *RPA* directly *in vitro*, and localizes to sites of DNA damage via *RPA* in cells. *PRP19* promotes ubiquitylation of *RPA* in a DNA damage-induced manner, and facilitates accumulation of *ATRIP* (ATR interacting protein) at the site of DNA damage. Depletion of *PRP19* compromises phosphorylation of *RPA32* and *CHK1*, leading to defective recovery of stalled replication forks and impaired fork progression on damaged DNA. Importantly, *PRP19* mutants that are unable to bind to *RPA* or function as an E3 ligase failed to support the *ATR* response, suggesting that full activation of *ATR* is driven by ubiquitylation-mediated circuitry orchestrated by *RPA*-ssDNA and *PRP19*. These results imply that the RNA-processing protein in undamaged cells transforms into a DNA damage sensor during DDR, revealing an unexpected interplay between these two fundamental processes [27].

Heterogeneous nuclear ribonucleoprotein U-like (*hnRNPUL*) proteins 1 and 2 have been identified as binding partners for the DNA double-strand break (DSB) sensor complex *MRE11*-*RAD50*-*NBS1* (MRN). The *hnRNPUL 1* and *2* proteins are recruited to DNA damage in an MRE11-dependent manner. They stimulate DNA-end resection and promote ATR-dependent signaling and DSB repair by HR. Furthermore, *hnRNPUL 1* and *2* function downstream of MRN and *CtIP* to promote recruitment of *BLM* (Bloom syndrome, RecQ helicase-like) helicase to DSBs [29,30]

We originally identified *RBM14* as a gene that radio-sensitizes glioblastoma multiforme (GBM) spheres when knocked down. *RBM14* is highly expressed in embryonic tissues and in stem cells, and has been implicated in RNA splicing as well as in transcription. It is thought that RBM14 controls transcription-coupled alternative splicing in a manner that depends on the promoter [31,32]. *RBM14* contains two N-terminal RNA recognition motifs (RRMs) and a prion-like domain (PLD). This PLD is found in oncoproteins *EWS* and *TLS*/*FUS* family proteins that are mutated both in human cancers and neurodegenerative diseases [33,34]. Amplification of *RBM14* has been observed in human cancers [35]. The RRM domains enable it to act as a regulator of mRNA splicing. The PLD is required for interaction with *TRBP* (thyroid-hormone-receptor-binding protein), *p300*, *SYT* (synovial sarcoma translocation protein), and *RUNX2*, and exhibits transcription regulation activity [36,37,38,39]. *RBM14* (also called *CoAA*) is alternatively spliced to produce a smaller protein called *CoAM* that lacks the PLD, which acts in a dominant negative fashion to regulate transcription [37,40]. Our results indicate that *RBM14* controls the non-homologous end-joining (NHEJ) pathway of DSB repair, and is involved in maintaining the stem-like state of GBM spheres. *RBM14* knockdown cells are sensitive to IR. *RBM14* interacts with *KU80*, and autophosphorylation of *DNA-PK* is compromised in *RBM14* knockdown cells. These results indicate that *RBM14* is required for the NHEJ pathway [41]. Most importantly, knockdown of *RBM14* reduces tumorigenicity and also radio-sensitizes GBM stem-like cells *in vivo* [41]. *RBM14* is a component of paraspeckles that are nuclear bodies composed of RBPs and the long noncoding RNA NEAT1 [42,43]. Interestingly, the other paraspeckle proteins, *FUS* and *NONO*, have also been implicated in DDR. *NONO* is recruited to laser-induced DSB sites in a PAR-dependent manner, and stimulates NHEJ and represses HR [44]. *FUS* is also recruited to laser-induced DSB sites in a PAR-dependent manner. However, knockdown of *FUS* inhibits both NHEJ and HR [45,46,47,48,49]. The function of *FUS* in DDR involves its direct interaction with histone deacetylase 1 (*HDAC1*). Knockdown of *FUS* abolishes *γH2AX* formation in response to DSBs [45], whereas *RBM14* knockdown induces prolonged *γH2AX* foci [41]. These results indicate that there might be several layers of controls to initiate a particular repair process by different PAR-recruited RBPs.

**Figure 1:**
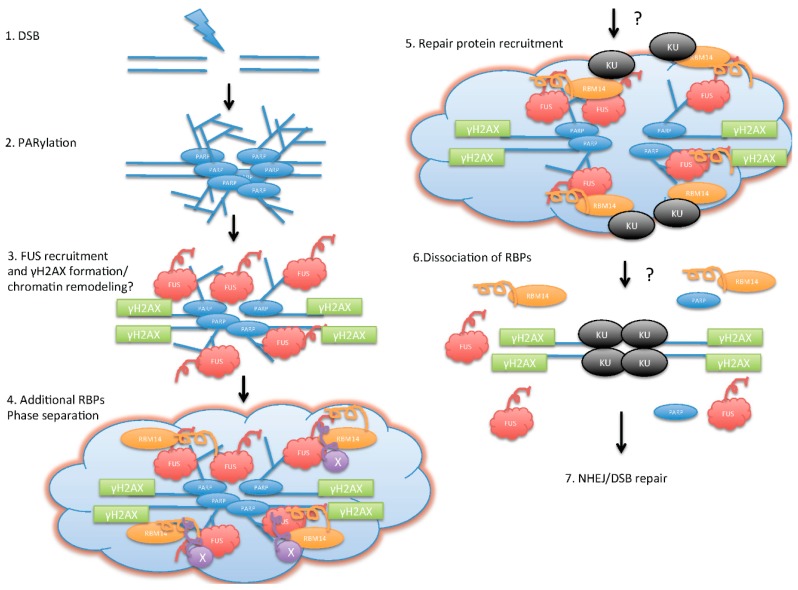
Illustration of model for poly(ADP-ribose) (PAR)/RNA-binding proteins (RBPs)-mediated initiation of DNA double-strand break (DSB) repair. Upon DSB formation (**1**); PARylation by *PARP* occurs around the DSB area, and creates platforms for RBPs (**2**); *FUS* (fused in sarcoma)proteins are recruited to the DSB sites in a PAR-dependent manner, and this step allows γH2AX formation (**3**); The additional RBPs with “prion”-like domains (PLDs) are recruited to the DSB sites, and form “functional” aggregation. This step starts initiation of phase separation/liquid-demixing (**4**); Phase separation creates an environment for the DSB repair protein recruitment (**5**); PAR degradation by *PARG* followed by dissociation of RBPs (**6**) allows DSB repair; (**7**) non-homologous end-joining (NHEJ)/DSB repair.

Like *FUS* and *NONO*, several other RBPs are recruited to laser-induced DSBs in a PAR- or PARP-dependent manner. These RBPs are intrinsically disordered proteins (IDPs) that contain an unstructured “prion”-like domain (PLD). PLDs are a subset of low complexity regions, enriched in unchanged polar amino acids and glycines, with similarities to the yeast prion protein [43,50]. Importantly, PLDs are often found in RBPs that drive protein aggregation in neurodegenerative disorders such as amyotrophic lateral sclerosis (ALS) [51]. These RBPs are detected on laser tracks within one minute after laser irradiation, and are excluded from the laser tracks shortly (within 10–15 min, depending on conditions of laser irradiation) [29,30,44,45,50]. It was shown recently that some of these RBPs, *FUS*, *EWS*, *TAF15*, and *RBM14*, form a PAR-dependent liquid-like compartment by phase separation at the laser-induced DSB sites [43,50]. This phase separation, also referred to as liquid demixing, requires the PLDs [43,50,51]. PLD-containing proteins/IDPs can phase-separate from the soluble intracellular space, and are disposed to aggregating under pathological conditions, forming “functional aggregates”. The nucleic acid–mimicking biopolymer PAR nucleates intracellular liquid demixing. PAR-levels are significantly increased at sites of DNA damage. PAR seeds RBPs/IDPs for liquid demixing, resulting in the rapid yet transient and fully reversible association of various RBPs/IDPs at DSB sites. Liquid-demixing relies on electrostatic interactions between positively charged RGG repeats (found in the PLDs) and negatively charged PAR, and is amplified by aggregation of PLDs. It has been proposed that PAR-seeded liquid demixing is a general mechanism to dynamically reorganize the soluble nuclear space. Deflected phase separation is implicated in pathological protein aggregation that is believed to cause neurological diseases [51]. However, it is not understood how these RBPs are involved in DNA repair pathways. This phase separation by PAR-seeded RBPs/IDPs is likely to provide orchestration of the earliest cellular DDR prior to the recruitment of classical “sensor” proteins (see Figure 1 for a model).

Immediate recruitment of prion-like RBPs to sites of DNA damage followed by phase separation requires PARP1. PARP1, which catalyzes the attachment of ADP-ribose units to target proteins, plays wide-ranging roles in cellular processes including DNA repair and transcription. *PARP1* is rapidly recruited to DNA damage sites including single-strand nicks and DSBs, where its catalytic activity is enhanced by up to orders of magnitude, resulting in the synthesis of protein-conjugated long branches of ADP-ribose chains [52,53]. The best-known roles of PARP1 are associated with DNA damage and genomic maintenance, with specific roles in base-excision/single-strand break repair. However, roles of *PARP1* in DSB repair remain controversial. *PARP1* is activated by DSBs, and is implicated in classical and alternative (c/alt) NHEJ and HR pathways [53]. *PARP1* is involved in the early recruitment of the MRN complex to DSBs [54], and interacts with cNHEJ components such as *KU* and *DNA-PK* [55,56]. However, *PARP1*-deficient cells do not exhibit cNHEJ defects [57]. Instead, biochemical studies suggest that *PARP1* facilitates altNHEJ and inhibits cNHEJ [58,59,60]. *PARP*-depleted cells show little effect on HR efficiency by homology-directed repair [61]. However, *PARP1* has been strongly implicated in recovery from stalled replication forks that is mediated by HR [57,62,63,64]. In fact, *PARP1* promotes recruitment of *MRE11* and *RAD51* specifically in response to stalled replication forks [54,65]. Understanding of PARylation/*PARP1*’s exact role in response to DNA damage requires further investigation. The PAR-dependent liquid-demixing seems to provide a platform for RBPs which contain PLDs (IDPs). This discovery is a new clue for exploring mechanisms of PAR-dependent initiation/selection of DDR pathways.

Mutations in proteins that contain the PLDs of low sequence complexity are associated with neurodegenerative and aging-associated diseases because the disordered sequences are prone to aggregation. *FUS* is a prion-like protein containing intrinsically disordered domains, and mutations in the *FUS* gene are implicated in causes of the neurodegenerative disease ALS. An *in vitro* “aging” experiment demonstrated that liquid droplets of *FUS* protein convert with time from a liquid to an aggregated state, and that this conversion is accelerated by patient-derived mutations. Therefore, the physiological role of *FUS* and presumably the other prion-like RBPs, the formation of dynamic liquid-like compartments through intrinsically disordered regions, faces trade-off between functionality and risk of pathological aggregations, causing ALS and, presumably, other age-related diseases [51].

## 4. Conclusions

Recent discovery of the “liquid-demixing” state created by PAR and IDPs at damaged DNA areas opened up a new paradigm in the field. Further investigation on how these prion-like domain-containing proteins orchestrate DNA repair pathways will deepen our understanding of neurological diseases and cancers caused by mutations in these proteins.
