# Kai, M. Roles of RNA-Binding Proteins in DNA Damage Response. *Int. J. Mol. Sci.* 2016, *17,* 310

**DOI:** 10.3390/ijms17040604

**Published:** 2016-04-21

**Authors:** Mihoko Kai

**Affiliations:** Department of Radiation Oncology, Johns Hopkins University, School of Medicine, Baltimore, MD 21231, USA; mkai2@jhmi.edu; Tel.: +1-410-614-9223

The author would like to insert the citation after the following sentence, “These RBPs are detected on laser trackswithin one minute after laser irradiation, and are excluded from the laser tracks shortly (within 10–15 min, depending on conditions of laser irradiation) [1]”, in the paper published in the *International Journal of Molecular Sciences* [2].

The author wants to stress that an important reference has been inadvertently omitted in the appended review. Britton *et al.* [1] recently documented the local exclusion from laser-or calicheamicin-generated DNA damage of the RNA binding protein SAF-A/hnRNP U together with several partners detected by mass-spectrometry, including TAF15 and FUS/TLS. The exclusion of this set of proteins is uncoupled from their initial poly(ADP-ribose)-dependent recruitment at these sites. In addition, using an original reporter for live imaging of R-loops (a catalytically inactive mutant of bacterial RNAseHI that binds with high affinity to *R*-loops), they presented several data supporting that the exclusion of these factors is part of a DDR Pi3K-like kinases-dependent mechanism operating at DNA damage sites to prevent R-loops accumulation.
